# State preparation in a Jaynes-Cummings lattice with quantum optimal control

**DOI:** 10.1038/s41598-023-47002-1

**Published:** 2023-11-14

**Authors:** Prabin Parajuli, Anuvetha Govindarajan, Lin Tian

**Affiliations:** School of Natural Sciences, University of California, Merced, California 95343 USA

**Keywords:** Quantum simulation, Superconducting devices, Quantum information

## Abstract

High-fidelity preparation of quantum states in an interacting many-body system is often hindered by the lack of knowledge of such states and by limited decoherence times. Here, we study a quantum optimal control (QOC) approach for fast generation of quantum ground states in a finite-sized Jaynes-Cummings lattice with unit filling. Our result shows that the QOC approach can generate quantum many-body states with high fidelity when the evolution time is above a threshold time, and it can significantly outperform the adiabatic approach. We study the dependence of the threshold time on the parameter constraints and the connection of the threshold time with the quantum speed limit. We also show that the QOC approach can be robust against control errors. Our result can lead to advances in the application of the QOC to many-body state preparation.

## Introduction

Recent progresses in manipulating quantum states and dynamics in noisy intermediate-scale quantum (NISQ) devices have demonstrated the potential to solve complicated problems with various physical platforms^1–3^. An important question among such problems is the preparation of many-body states with high fidelity using NISQ devices, which is crucial for quantum simulation, quantum metrology and quantum communication^4–7^. In the past, a number of approaches have been developed to generate desired quantum many-body states, including adiabatic processes^8,9^, quantum shortcut approach^10^, quantum phase estimation ^11,12^, quantum eigensolvers^13–15^, and open system approach^16,17^. However, due to the intrinsic complexity of quantum many-body systems, it remains challenging to prepare such states with high accuracy.

With quantum control techniques, precisely engineered pulse sequences have been employed to manipulate quantum states with high accuracy^18^. Among such techniques, the quantum optimal control (QOC) approach^19–22^ provides a computational framework to generate desired quantum states or quantum processes by searching for optimal, time-dependent control parameters under given constraints. In recent years, QOC has been widely used in a broad range of applications from the implementation of high-fidelity quantum logic gates, the suppression of environmental noise, the control of quantum transduction processes, the generation of novel entangled states, to the control of quantum many-body systems^23–27^. The problem of preparing quantum states or processes can be formulated into an optimization problem in the QOC approach, where an algorithm is adopted to minimize the cost function.

Here, we study the QOC approach for the preparation of many-body states in a finite-sized Jaynes-Cummings (JC) lattice. In the thermodynamic limit, a JC lattice with integer fillings (i.e., the average number of excitations per lattice site is an integer) can exhibit a quantum phase transition between the Mott-insulating (MI) and superfluid (SF) phases^28–33^. At a finite size, the ground states of a JC lattice in the MI and SF regimes still exhibit distinctive behaviors^34–36^. The preparation of the ground states in a JC lattice is non-trivial, especially in the intermediate range between the deep MI and deep SF phases. In^37^, we employed an optimized nonlinear adiabatic approach for state preparation in a JC lattice. In this work with the QOC approach, we adopt the chopped random basis (CRAB) algorithm^38,39^ to parameterize the time-dependent couplings of the JC lattice and use the Nelder-Mead approach to optimize these couplings. Our numerical result shows that when the total evolution time is above a threshold time $T_{\textrm th}$, the QOC approach can generate the target state with a high fidelity above a designated threshold value, and it can significantly outperform the adiabatic approach. We find that the threshold time decreases and the average energy fluctuation increases with the constraints on the time-dependent couplings, which indicates the connection between the threshold time and quantum speed limit (QSL)^40–45^. Furthermore, our numerical simulation shows that the QOC approach can be robust against control errors in the time-dependent couplings. JC lattices have been explored theoretically and implemented experimentally in various systems, including the circuit QED systems, nanophotonic devices, atoms, and trapped ions^46–57^. This work can shed light on the application of QOC in many-body state preparation and lead to deeper understanding of the QSL for preparing quantum many-body states.

## Results

### JC lattice

**Figure 1:**
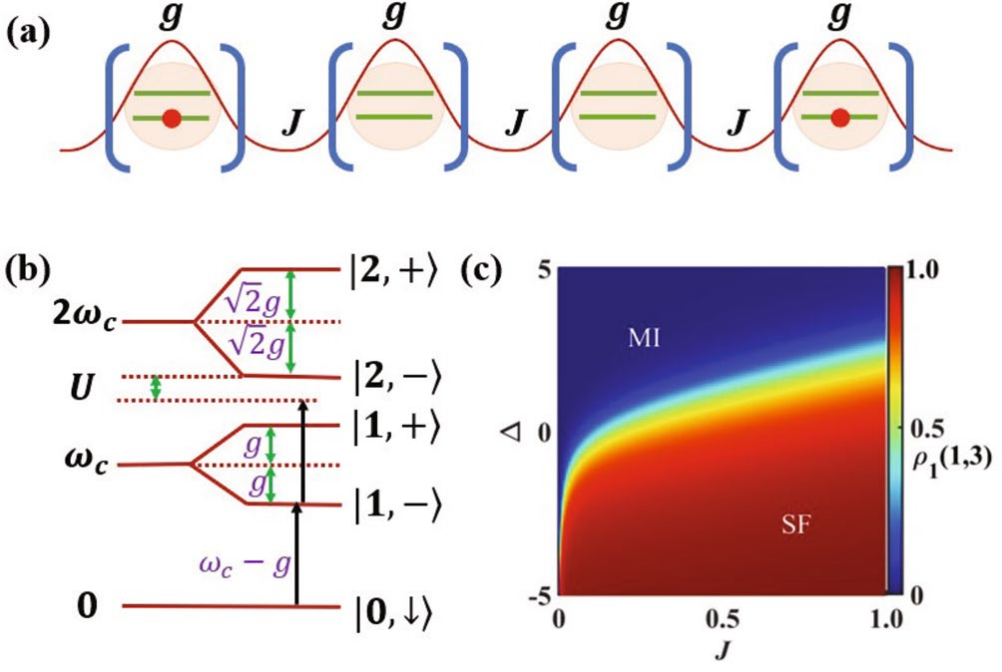
Jaynes-Cummings Lattice. (**a**) The schematic of a one-dimensional JC lattice with qubit-cavity coupling *g* and photon hopping rate *J*. (**b**) The energy spectrum of a single JC model for detuning $\Delta =0$, where $\vert 0,\downarrow \rangle$ is the ground state and $\vert n,\pm \rangle$ ($n\geq1$, integer) are the lowest excited states. (**c**) The single particle density matrix $\rho _{1}(1,3)$ vs hopping rate *J* and detuning $\Delta$ for a $N=4$ lattice at unit filling. Here we let $g=1$, and all parameters are in dimensionless units^58^.

A JC lattice is illustrated in Fig. 1a, where each unit cell of the lattice contains a two-level system (qubit) coupled to a cavity mode with coupling strength *g*, and adjacent cavities are coupled by photon hopping with hopping rate *J*. The Hamiltonian of this lattice can be written as $H_t =H_0 +H_{\textrm int}$ ($\hbar = 1$). Here

1 $$\begin{aligned} H_0=\sum _{j=1}^N\left[ \omega _c a_j^\dag a_j +\omega _{z}\frac{\sigma _{jz}+1}{2} + g\left( a_{j}^{\dagger }\sigma _{j-}+\sigma _{j+}a_j\right) \right] \end{aligned}$$

is the Hamiltonian of the JC models in a finite-sized lattice of size *N*, with $j\in [1, N]$, $\omega _c$ the cavity frequency, $a_j$ ($a_j^\dagger$) the annihilation (creation) operator of the cavity modes, $\omega _{z}$ the energy splitting of the qubits, and $\sigma _{jz}, \sigma _{j\pm }$ the Pauli operators of the qubits. Also,

2 $$\begin{aligned} H_{\textrm int}=-J\sum _{j=1}^N\left( a_{j}^{\dagger }a_{j+1}+a_{j+1}^{\dagger }a_{j}\right) \end{aligned}$$

describes photon hopping between neighboring sites in the lattice. We choose the periodic boundary condition with $a_{N+1}=a_1$ and denote $\Delta =\omega _{c}-\omega _{z}$ as the detuning between cavity and qubit frequencies.

The qubit-cavity coupling *g* induces a built-in nonlinearity in the energy spectrum of a single JC model^59^, which can be viewed as an effective onsite interaction with strength *U*, as shown in Fig. 1b. Details of the JC model spectrum can be found in the Supplementary Information. In the thermodynamic limit with $N\rightarrow \infty$, and at integer fillings when the number of excitations is an integer multiple of *N*, the competition between this onsite interaction and the photon hopping can lead to a quantum phase transition between the MI and SF phases ^28–31^. When dominated by the qubit-cavity coupling with $g\gg J$, the ground state of the JC lattice will be in a MI phase characterized by localized polariton excitations. In the limiting case of $J=0$, the ground state with *N* excitations is the product state $\vert G\rangle _{J=0}=\prod _{j=1}^{N} \vert 1,-\rangle _j$ with each JC model in its first excited state $\vert 1,-\rangle _j$. When dominated by photon hopping with $J\gg g$, the ground state of the lattice will be in a SF phase with long range correlation. In the limiting case of $g=0$, the ground state is the Fock state $\vert G\rangle _{g=0} = \frac{1}{\sqrt{N!}} (a_{k=0}^\dagger )^N \vert 0,\downarrow \rangle$ with all excitations occupying the momentum-space mode $a_{k=0}=\frac{1}{\sqrt{N}}\sum _{j=1}^{N} a_j e^{i k\cdot j}$ for the quasi-momentum $k=0$. For a finite-sized lattice, the ground states also exhibit features of these phases in the corresponding parameter regimes^35,37^. These features can be illustrated with the single-particle density matrix $\rho _{1} (i,j)=\langle G|a_{i}^{\dagger }a_{j}|G\rangle /\langle G|a_{i}^{ \dagger }a_{i}|G\rangle$, which describes the spatial correlation between the cavity modes at sites *i* and *j*, with $\vert G\rangle$ the ground state for given parameters. As shown in Fig. 1c, $\rho _{1} (1,3)$ for a $N=4$ lattice, and hence, the spatial correlation of the ground state, decreases algebraically (exponentially) in the SF (MI) phase.

### Couplings and fidelity

Preparing the ground states of a JC lattice with integer fillings is a challenging task except for the limiting cases of $g=0$ or $J=0$. Here, we will employ the QOC technique to achieve fast and high-fidelity state preparation in a JC lattice and compare our result with that of the adiabatic approach in^37^. We define the fidelity of the prepared state with regard to the desired many-body ground state $\vert \psi _{\textrm T}\rangle$ for the target parameters as

3 $$\begin{aligned} {\mathbb {F}}= \vert \langle \psi (T)|\psi _{\textrm T}\rangle \vert ^{\textrm 2}, \end{aligned}$$

where $\vert \psi (T)\rangle$ is the state at the final time *T* of the evolution. The cost function in the QOC is chosen as the infidelity ${\mathbb {I}}=1-{\mathbb {F}}$. The QOC approach minimizes the cost function by optimizing the coupling constants $g\left( t \right)$ and $J\left( t\right)$ in the Hamiltonian $H_t$^19–22^. For simplicity of discussion, we let the detuning $\Delta (t)\equiv 0$ during the entire evolution. The couplings are bounded by the constraints $g_{\textrm max}$ and $J_{\textrm max}$, with $\vert g(t)\vert \le g_{\textrm max}$ and $\vert J(t)\vert \le J_{\textrm max}$ at an arbitrary time *t*. The numerical simulation is conducted on a JC lattice with four sites and four polariton excitations (i.e., unit filling). The initial Hamiltonian parameters are $g(0)=0$ and $J(0)=0.5$, and the target parameters are $g(T)=1$ and $J(T)=0.02$. The initial state of this system is the ground state for the initial parameters, which is the SF state $\vert G\rangle _{g=0}$. The target state is the ground state for the target parameters, which is a MI state. During the evolution, the system is governed by the Hamiltonian $H_t$ with time-dependent couplings *g*(*t*) and *J*(*t*). We adopt the CRAB algorithm that parameterizes the couplings with truncated Fourier series^38,39^, and apply the Nelder-Mead method for the optimization.

**Figure 2:**
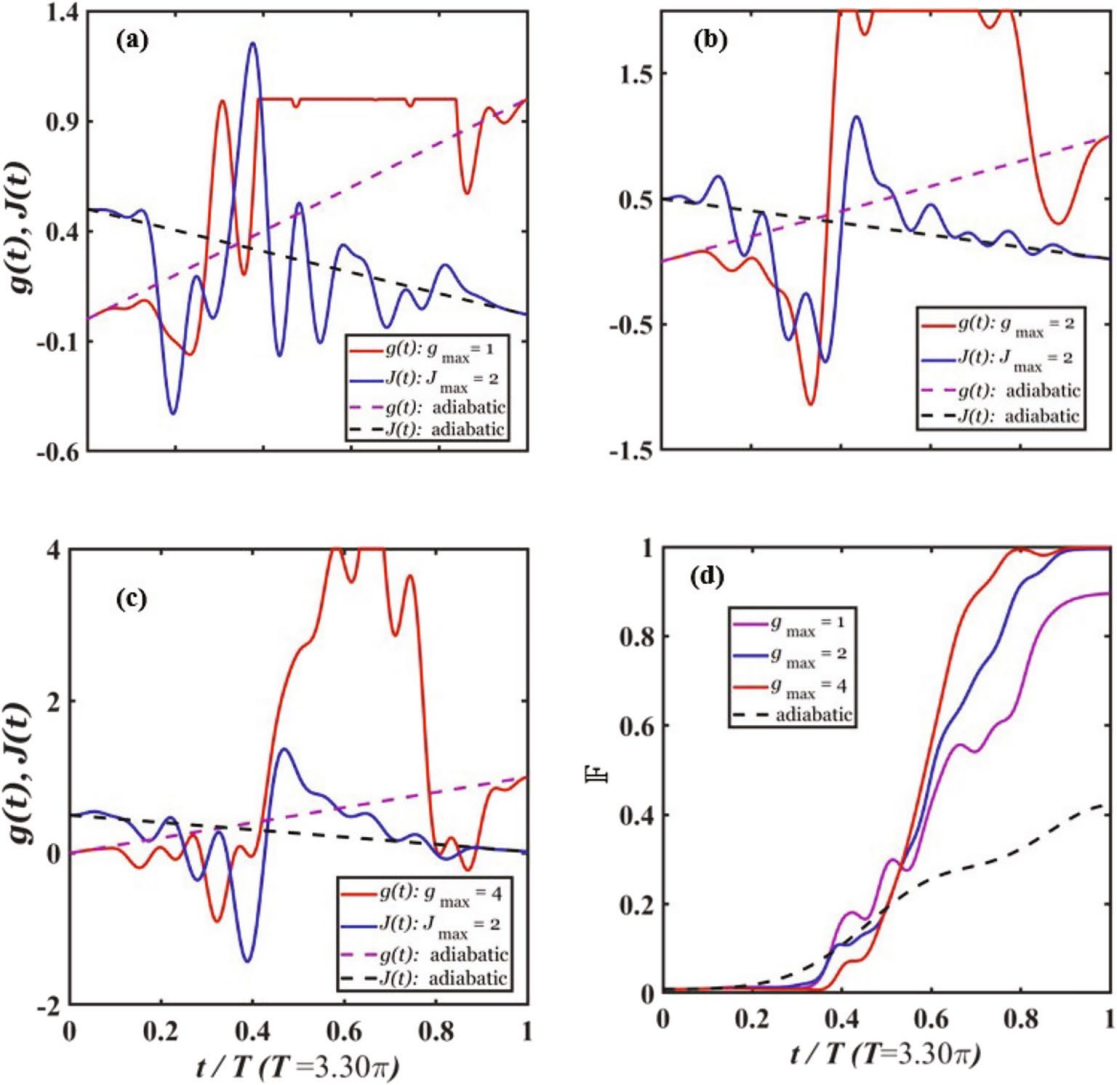
Optimized couplings. (a–c) The optimized couplings *g*(*t*) and *J*(*t*) vs the relative evolution time *t*/*T* for the constraints $J_{\textrm max}=2$ and $g_{\textrm max}=1, 2, 4$, respectively, with the total evolution time $T=3.30\pi$. **d** The fidelity ${\mathbb {F}}$ vs *t*/*T* for the couplings in (**a**–**c**). The dashed curves are for the adiabatic ramping process.

In Fig. 2a–c, we plot the optimized couplings *g*(*t*) and *J*(*t*) vs the relative evolution time *t*/*T* under the constraints $J_{\textrm max}=2$ and $g_{\textrm max}=1, 2, 4$, respectively, with total evolution time $T=3.30\pi$. The couplings in the adiabatic approach governed by (8a) and (8b) are plotted as dashed curves. The optimized couplings are continuous curves that change smoothly over the course of the evolution. For the constraint $g_{\textrm max}=1$, *g*(*t*) includes a large plateau at the maximal strength $g(t)=1$; whereas the plateau area decreases significantly for $g_{\textrm max}=4$. In contrast, *J*(*t*) has no plateau. This is because the system can already reach the deep SF phase when $J=J_{\textrm max}=1$, and it does not require a larger value of *J* to explore the SF part of the Hilbert space. Our numerical result also shows that the fidelity for larger $g_{\textrm max}$ is significantly higher than that for smaller $g_{\textrm max}$, as shown in Fig. 2d. For $g_{\textrm max}=2$ and 4, the fidelity of the state at the final time exceeds the designated threshold fidelity ${\mathbb {F}}_{\textrm th}=0.99$; while for $g_{\textrm max}=1$, the fidelity cannot reach 0.99 after the maximal number of iterations. For all the three $g_{\textrm max}$ values, the fidelity at time *T* is much higher than that from the adiabatic approach, demonstrating that the QOC approach can greatly outperform the adiabatic approach.

**Figure 3:**
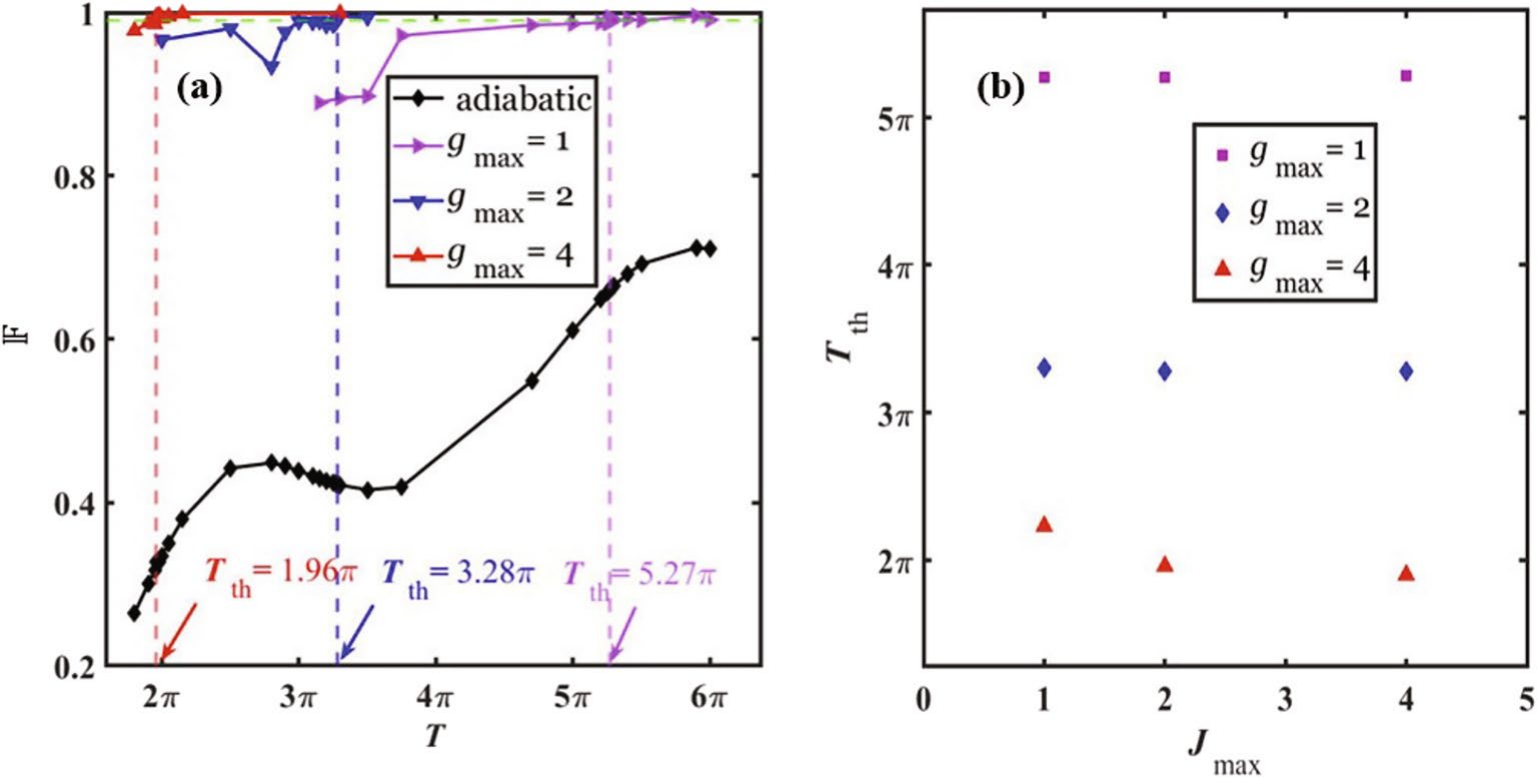
Fidelity and threshold time. (a) The fidelity ${\mathbb {F}}$ of the prepared state vs the total evolution time *T*. The vertical dashed lines indicate the position of the threshold time $T_{\textrm th}$ for each set of constraints. The constraints are $J_{\textrm max}=2$ and $g_{\textrm max}=1, 2, 4$. The dashed horizontal line corresponds to the threshold fidelity ${\mathbb {F}}_{\textrm th}=0.99$. (**b**) The threshold time $T_{\textrm th}$ vs the constraint $J_{\textrm max}$ for $g_{\textrm max}=1, 2, 4$.

The fidelity of the prepared state depends on the constraints $g_{\textrm max}$, $J_{\textrm max}$, and the total evolution time *T*. In Fig. 3a, we plot the fidelity vs *T* for the constraints $g_{\textrm max}=1, 2, 4$ and $J_{\textrm max}=2$. The result shows that the fidelity exhibits an increasing trend with the total time *T* and the constraint $g_{\textrm max}$. Meanwhile, the fidelity from the QOC approach is significantly higher than the fidelity from the adiabatic ramping process. For example, the QOC fidelity is greater than 0.99 for $g_{\textrm max}=2$, $J_{\textrm max}=2$ and $T= 3.30\pi$, while the fidelity from the adiabatic ramping is only 0.42 for the same parameter constraints and evolution time *T*.

### Threshold time

In the numerical simulation, we observe that when the total evolution time *T* is below a threshold time $T_{\textrm th}$, the QOC process cannot achieve a fidelity that is higher than the designated threshold fidelity, which we choose to be ${\mathbb {F}}_{\textrm th}=0.99$. In Fig. 3a, the threshold time $T_{\textrm th}$ for each set of constraints is indicated by a dashed vertical line. Our result shows that the threshold time decreases as the constraint $g_{\textrm max}$ increases. Hence, it will take less time to reach a desired fidelity when the coupling *g*(*t*) can have a larger magnitude. To analyze the dependence of the threshold time on the constraints, we plot $T_{\textrm th}$ vs the constraint $J_{\textrm max}$ for different values of $g_{\textrm max}$ in Fig. 3b. It is shown that $T_{\textrm th}$ decreases significantly as $g_{\textrm max}$ increases, but only decreases slightly when $J_{\textrm max}$ increases. For the values of $g_{\textrm max}$ used in our simulation, $J=J_{\textrm max}=1$ is sufficiently large for the system to enter the deep SF phase. Thus, the system does not demand a larger value of *J* or subsequently longer evolution time in order to reach high fidelity, which leads to $T_{\textrm th}$’s weak dependence on $J_{\textrm max}$. This result agrees with that of Fig. 2a–c, where *J*(*t*) does not exhibit any plateau during the evolution. The threshold time for different constraints is given in Table 1, together with the fidelity at the evolution time $T=T_{\textrm th}$ from the QOC approach and from adiabatic ramping.

**Table 1:** The threshold time $T_{\textrm th}$ for selected constraints and the corresponding fidelity ${\mathbb {F}}$ at the total evolution time $T=T_{\textrm th}$ using QOC and using adiabatic ramping (adia.).

Constraints	$T_{\textrm th}$	${\mathbb {F}}$ (QOC)	${\mathbb {F}}$ (adia.)
$J_{\textrm max}=1, g_{\textrm max}=1$	$5.27\pi$	0.9944	0.6610
$J_{\textrm max}=1, g_{\textrm max}=2$	$3.30\pi$	0.9932	0.4213
$J_{\textrm max}=1, g_{\textrm max}=4$	$2.23\pi$	0.9963	0.3995
$J_{\textrm max}=2, g_{\textrm max}=1$	$5.27\pi$	0.9944	0.6610
$J_{\textrm max}=2, g_{\textrm max}=2$	$3.28\pi$	0.9927	0.4223
$J_{\textrm max}=2, g_{\textrm max}=4$	$1.96\pi$	0.9954	0.3276
$J_{\textrm max}=4, g_{\textrm max}=1$	$5.28\pi$	0.9925	0.6626
$J_{\textrm max}=4, g_{\textrm max}=2$	$3.28\pi$	0.9927	0.4223
$J_{\textrm max}=4, g_{\textrm max}=4$	$1.90\pi$	0.9904	0.3001

We compare the threshold time $T_{\textrm th}$ from our numerical simulation with an estimation of the quantum speed limit (QSL) $T_{\textrm QSL}$, which is the minimal time for a given quantum system to evolve from an initial state to a target state^40–45^. We estimate the QSL with^43^:

4 $$\begin{aligned} T_{\textrm QSL}\approx \frac{\arccos {\left| \langle \psi (0)\vert \psi _{\textrm T}\rangle \right| }}{\Delta E_{\textrm ave}}. \end{aligned}$$

Here, $\arccos {\left| \langle \psi (0)\vert \psi _{\textrm T}\rangle \right| }$ describes the distance between the initial and the target states in the Hilbert space^45^. For orthogonal states with $\langle \psi (0)\vert \psi _{\textrm T}\rangle =0$, the distance is $\pi /2$. For the initial and target states in our simulation, the distance is $0.469 \pi$. Also, $\Delta E_{\textrm ave} = \frac{1}{T} \int _0^T dt \Delta E (t)$ is the average energy fluctuation during the time evolution with $\Delta E (t)=\sqrt{\langle [H_t - \langle H_t\rangle ]^2 \rangle }$ being the instantaneous energy fluctuation of the Hamiltonian $H_t$ at time *t*, and the operator average is taken on the instantaneous quantum state $\vert \psi (t)\rangle$.

**Figure 4:**
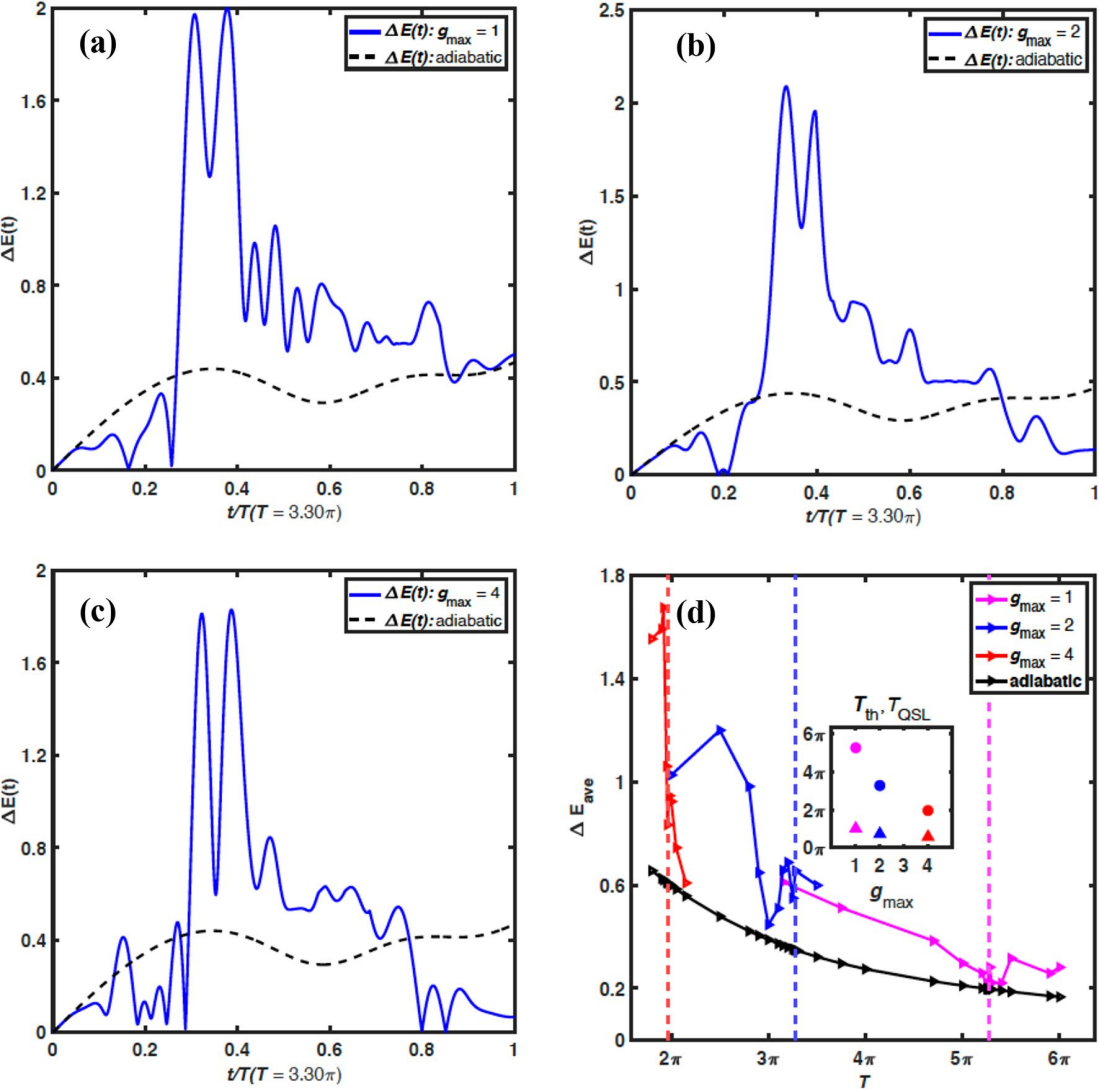
Energy fluctuation. (**a**–**c**) The energy fluctuation $\Delta E(t)$ vs the relative evolution time *t*/*T* for the constraints $J_{\textrm max}=2$ and $g_{\textrm max}=1, 2, 4$, respectively, and $T=3.30\pi$. The dashed curve is for the adiabatic ramping process. (**d**) The average energy fluctuation $\Delta E_{\textrm ave}$ vs the total evolution time *T* for the constraints in (**a**–**c**). The inset of (**d**) shows the threshold time $T_{\textrm th}$ (circle) and the estimated $T_{\textrm QSL}$ (triangle) vs $g_{\textrm max}$.

In Fig. 4a–c, we plot the energy fluctuation $\Delta E(t)$ as a function of the relative evolution time *t*/*T* for the same constraints $J_{\textrm max}$, $g_{\textrm max}$, and the same evolution time *T* as those in Fig. 2. The result from the adiabatic approach is plotted as the dashed curve. In all the three plots, the energy fluctuation is the strongest when $t/T\in (0.3,\,0.4)$, and it is far stronger than the energy fluctuation in the adiabatic process. For $g_{\textrm max}=2, 4$, $\Delta E(t)$ becomes very small when *t* approaches the final time *T*, indicating that the final state occupies the ground state with high probability. For $g_{\textrm max}=1$, $\Delta E(t)$ at $t=T$ remains large and is comparable to that from the adiabatic approach, which shows that the system has a sizable probability to be in the excited states in this case. This is because the threshold time for $g_{\textrm max}=2, 4$ (for $g_{\textrm max}=1$) is shorter (longer) than the evolution time $T=3.30\pi$, and hence the QOC process can (cannot) reach high fidelity. In Fig. 4d, we plot the average fluctuation energy $\Delta E_{\textrm ave}$ vs the total evolution time *T* for the constraints used in Fig. 4a–c. Here $\Delta E_{\textrm ave}$ shows a decreasing trend as *T* increases and is stronger than that from the adiabatic approach. Using (4) and the result of $\Delta E_{\textrm ave}$ for the threshold time $T_{\textrm th}$, we estimate the QSL. As shown in the inset of Fig. 4d, the estimated $T_{\textrm QSL}$ exhibits similar behavior to the threshold time $T_{\textrm th}$, decreasing with the increase of the constraint $g_{\textrm max}$. Meanwhile, the estimated QSL is comparable in scale to the threshold time, but it is shorter than the threshold time. We note that this comparison is only qualitative. The estimation of the QSL presented here is a rough approximation due to the complexity of the JC lattice, and the threshold time is defined for a specific threshold fidelity chosen in our numerical simulation.

## Discussion

### Control error and decoherence

In superconducting quantum devices, tunable qubit-cavity coupling and cavity hopping (i.e., cavity coupling) can reach a few hundreds of MHz^60–63^. For example, tunable qubit-cavity coupling can be achieved via flux-tuned inductive coupling in the g-mon configuration or via a tunable coupler^64,65^. Tunable cavity hopping can be achieved by connecting cavities with a tunable Josephson junction^66^. We assume that the dimensionless coupling $g=1$ used in our numerical simulation corresponds to $g=2\pi \times 100$ MHz in actual devices^58^. A dimensionless evolution time of $T=3.30\pi$ then corresponds to $T=16.5\,$ ns. The optimized, time-dependent couplings *g*(*t*) and *J*(*t*) need to be generated within this time scale, which can be implemented with current technology.

To explore the robustness of the QOC approach against control errors, we simulate the errors by adding a time-dependent Gaussian noise to the optimized solutions of *g*(*t*) and *J*(*t*) with

5a $$\begin{aligned} g(t)&\rightarrow g(t)+\delta _{1}(t), \end{aligned}$$

5b $$\begin{aligned} J(t)&\rightarrow J(t)+\delta _{2}(t), \end{aligned}$$

where $\delta _{1}(t)$ and $\delta _{2}(t)$ are Gaussian noise at time *t* with standard deviation $\sigma$. We obtain the fidelity of the prepared state in the presence of these errors. For a given value of $\sigma$, we conduct the simulation on 1000 samples of the time-dependent errors and calculate the average value of the fidelity. The total evolution time is chosen to be the threshold time $T_{\textrm th}$ for given constraints.

**Figure 5:**
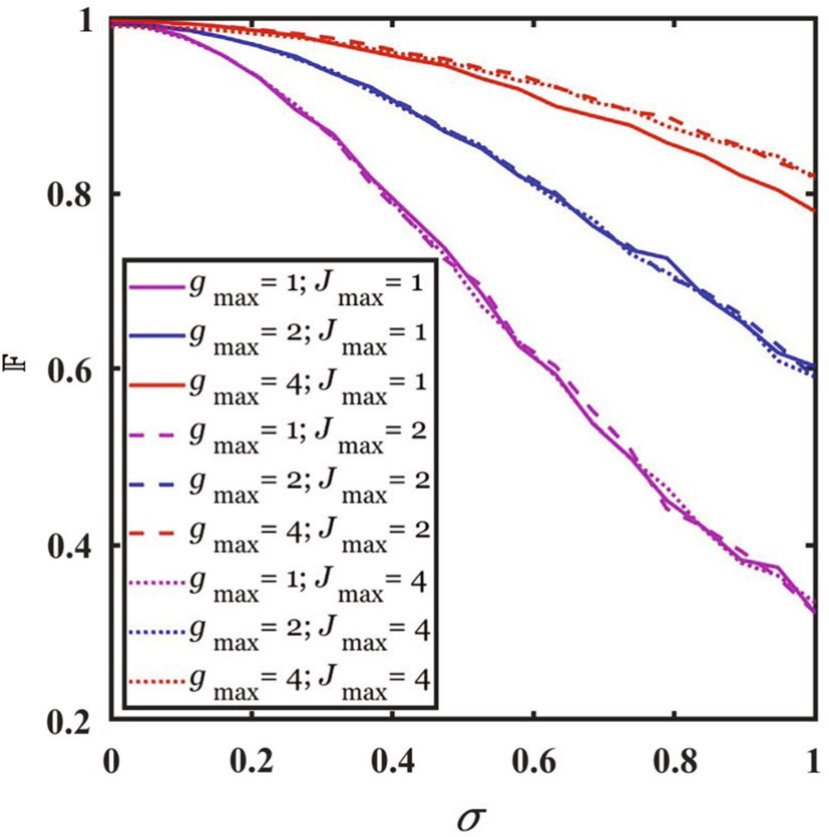
Effect of control error. The fidelity vs the standard deviation $\sigma$ of Gaussian control errors. The total evolution time for given constraints is the corresponding threshold time $T_{\textrm th}$ in Table 1.

Figure 5 shows that the fidelity of the prepared state decreases with the standard deviation of the control errors. We observe that the decrease of the fidelity for larger $g_{\textrm max}$ is slower than that for smaller $g_{\textrm max}$; whereas the decrease of the fidelity remains almost the same for different values of $J_{\textrm max}$. For $J_{\textrm max}=2$ and $\sigma =0.05$, the fidelity is reduced to ${\mathbb {F}}=0.9902$, 0.9910, 0.9948 for $g_{\textrm max}=1$, 2, 4, respectively. Compared with the fidelity for no control errors given in Table 1, the reduction of the fidelity is negligible. This result shows that the QOC approach can be robust against control error.

Another factor that could affect the fidelity of the QOC process is the decoherence of the qubits and the cavity modes. In the NISQ era, the finite decoherence times of the quantum devices set a limit on the time for coherent evolution. In a JC lattice with unit filling, the polariton excitations can decay in a time scale comparable to the decoherence times. At the current state-of-the-art, the decoherence time of superconducting qubits can reach $\sim 100$ μs ^60–62^. Superconducting cavities can have quality factors greater than $10^6$. For a cavity frequency of $\omega _c=2\pi \times 5$ GHz, such a quality factor gives a cavity decay time of ~ 32 μs. With an evolution time of 16.5 ns, the QOC can be completed in a much shorter time scale than the decoherence times of superconducting qubits and cavities, and hence the effect of decoherence can be neglected. This analysis has been confirmed by our numerical simulation using a master equation approach, as detailed in the Supplementary Information. We want to note that the short evolution time required in the QOC approach is one of its advantages over the adiabatic approach.

## Methods

In the CRAB algorithm used in our simulation, the time-dependent parameters *g*(*t*) and *J*(*t*) are parametrized with truncated Fourier series to the 8th harmonics and can be written as^38,39^

6a $$\begin{aligned} g(t)&=g_{0}(t)\left[ 1 +s(t)f_1(t)\right] , \end{aligned}$$

6b $$\begin{aligned} J(t)&=J_{0}(t)\left[ 1 +s(t) f_2(t) \right] , \end{aligned}$$

with

7a $$\begin{aligned} f_1(t)&= \sum _{k=1}^{8} c_{1,k} \cos \left( \frac{\omega _{1,k}t}{T}\right) +c_{2,k} \sin \left( \frac{\omega _{1,k}t}{T}\right) , \end{aligned}$$

7b $$\begin{aligned} f_2(t)&= \sum _{k=1}^{8} d_{1,k} \cos \left( \frac{\omega _{2,k}t}{T}\right) +d_{2,k} \sin \left( \frac{\omega _{2,k}t}{T}\right) , \end{aligned}$$

where $c_{i,k}$ and $d_{i,k}$ ($i=1,\,2$ and $k\in [1,8]$) are the Fourier coefficients of the *k*-th harmonic in *g*(*t*) and *J*(*t*), respectively, and $\omega _{i,k}=k+\delta \omega _{i,k}$ is the frequency of the *k*-th harmonics with an adjustable offset $\delta \omega _{i,k}$. Here, $g_{0}(t)$ [$J_{0}(t)$] is the linear ramping function for the coupling *g* (*J*) in the adiabatic approach with

8a $$\begin{aligned} g_{0}(t)&=g(0)+ \left[ g(T)-g(0)\right] t/T, \end{aligned}$$

8b $$\begin{aligned} J_{0}(t)&=J(0)+\left[ J(T)-J(0)\right] t/T, \end{aligned}$$

where *g*(0), *J*(0) [*g*(*T*), *J*(*T*)] are the initial (target) values for the couplings. The function $s(t)=\left[ 1-\cos (2\pi t/T)\right]$. With $s(0)=s(T)=0$, it ensures that the initial and final values of *g*(*t*) [*J*(*t*)] are the same as that of $g_0(t)$ [$J_0(t)$]. During the QOC process, *g*(*t*) is bounded by the constraint $g_{\textrm max}$ with

9 $$\begin{aligned} g(t) = {\left\{ \begin{array}{ll} g(t), &{} \text {if\quad } \vert g(t)\vert \le g_{\textrm max} \\ g_{\textrm max} \frac{g(t)}{\vert g(t)\vert }, &{} \text {if\quad } \vert g(t)\vert > g_{\textrm max}; \end{array}\right. } \end{aligned}$$

and similarly, *J*(*t*) is bounded by the constraint $J_{\textrm max}$.

For a given set of initial (target) parameters for the JC lattice, we obtain the initial state $\vert \psi _0\rangle$ (the target state $\vert \psi _{\textrm T}\rangle$) by diagonalizing the corresponding Hamiltonian $H_{t}$. The optimization process begins with a random set of parameters $c_{i,k}$, $d_{i,k}$, and $\delta \omega _{i,k}$ and has a maximum of 150,000 iterations. The convergence of these parameters vs the iteration number *n* can be found in Fig. S1 of the Supplementary Information.

### Supplementary Information

Supplementary Information.

## Data Availability

The data that support the findings of this study are available from the corresponding author upon reasonable request.

## Code availability

The codes that are used to produce the data presented in this study are available from the corresponding author upon reasonable request.

## References

[1] Preskill J (2018). Quantum Computing in the NISQ era and beyond. Quantum.

[2] Arute F (2019). Quantum supremacy using a programmable superconducting processor. Nature.

[3] Zhong H-S (2020). Quantum computational advantage using photons. Science.

[4] Altman E (2021). Quantum simulators: Architectures and opportunities. PRX Quantum.

[5] Noh C, Angelakis DG (2017). Quantum simulations and many-body physics with light. Rep. Prog. Phys..

[6] Degen CL, Reinhard F, Cappellaro P (2017). Quantum sensing. Rev. Mod. Phys..

[7] Wei SH (2022). Towards real-world quantum networks: A review. Laser Photonics Rev..

[8] Farhi E (2001). A quantum adiabatic evolution algorithm applied to random instances of an NP-complete problem. Science.

[9] Albash T, Lidar DA (2018). Adiabatic quantum computation. Rev. Mod. Phys..

[10] Guéry-Odelin D, Raschhaupt A, Kiely A, Torrontegui E, Martínez-Garraot S, Muga JG (2019). Shortcuts to adiabaticity: Concepts, methods, and applications.. Rev. Mod. Phys..

[11] Kitaev, A. Y. Quantum measurements and the Abelian stabilizer problem. arXiv:quant-ph/9511026.

[12] Abrams DS, Lloyd S (1997). Simulation of many-body Fermi systems on a universal quantum computer. Phys. Rev. Lett..

[13] Peruzzo A (2014). A variational eigenvalue solver on a photonic quantum processor. Nat. Commun..

[14] Dumitrescu EF (2018). Cloud quantum computing of an atomic nucleus. Phys. Rev. Lett..

[15] Wei SJ, Li H, Long GL (2020). A full quantum Eigensolver for quantum chemistry simulations. Research.

[16] Kraus B (2008). Preparation of entangled states by quantum Markov processes. Phys. Rev. A.

[17] Verstraete F, Wolf MM, Cirac JI (2009). Quantum computation & quantum-state engineering driven by dissipation. Nat. Phys..

[18] D’Alessandro D (2008). Introduction of Quantum Control and Dynamics.

[19] Krotov VF (1996). Global Methods in Optimal Control Theory.

[20] Peirce AP, Dahleh MA, Rabitz H (1988). Optimal control of quantum-mechanical systems: Existence, numerical approximation, and applications. Phys. Rev. A.

[21] Werschnik J, Gross EKU (2007). Quantum optimal control theory. J. Phys. B At. Mol. Opt. Phys..

[22] Magann AB (2021). From pulses to circuits and back again: A quantum optimal control perspective on variational quantum algorithms. PRX Quantum.

[23] Khaneja N, Reiss T, Kehlet C, Schulte-Herbrueggen T, Glaser SJ (2005). Optimal control of coupled spin dynamics: Design of NMR pulse sequences by gradient ascent algorithms. J. Magn. Reson..

[24] Koch CP (2022). Quantum optimal control in quantum technologies. Strategic report on current status, visions, and goals for research in Europe. EPJ Quantum Technol..

[25] Head-Marsden K, Flick J, Ciccarino CJ, Narang P (2021). Quantum information and algorithms for correlated quantum matter. Chem. Rev..

[26] Bharti K (2022). Noisy intermediate-scale quantum algorithms. Rev. Mod. Phys..

[27] Doria P, Calarco T, Montangero S (2011). Optimal control technique for many-body quantum dynamics. Phys. Rev. Lett..

[28] Hartmann MJ, Brandão FGSL, Plenio MB (2006). Strongly interacting polaritons in coupled arrays of cavities. Nat. Phys..

[29] Greentree AD, Tahan C, Cole JH, Hollenberg LCL (2006). Quantum phase transitions of light. Nat. Phys..

[30] Angelakis DG, Santos MF, Bose S (2007). Photon-blockade-induced Mott transitions and XY spin models in coupled cavity arrays. Phys. Rev. A.

[31] Rossini D, Fazio R (2007). Mott-insulating and glassy phases of polaritons in 1D arrays of coupled cavities. Phys. Rev. Lett..

[32] Na N, Utsunomiya S, Tian L, Yamamoto Y (2008). Strongly correlated polaritons in a two-dimensional array of photonic crystal microcavities. Phys. Rev. A.

[33] Noh C, Angelakis DG (2017). Quantum simulations and many-body physics with light. Rep. Prog. Phys..

[34] Hu Y, Tian L (2011). Deterministic generation of entangled photons in superconducting resonator arrays. Phys. Rev. Lett..

[35] Seo K, Tian L (2015). Quantum phase transition in a multiconnected superconducting Jaynes-Cummings lattice. Phys. Rev. B.

[36] Seo K, Tian L (2015). Mott insulator-superfluid phase transition in a detuned multi-connected Jaynes-Cummings lattice. Sci. China Phys. Mech. Astron..

[37] Cai K, Parajuli P, Long G-L, Wong C-W, Tian L (2021). Robust preparation of many-body ground states in Jaynes-Cummings lattices. Npj Quantum Inf..

[38] Caneva T, Calarco T, Montangero S (2011). Chopped random-basis quantum optimization. Phys. Rev. A.

[39] Müller MM, Said RS, Delezko F, Calarco T, Montangero S (2022). One decade of quantum optimal control in the chopped random basis. Rep. Prog. Phys..

[40] Bhattacharyya K (1983). Quantum decay and the Mandelstam-Tamm-energy inequality. J. Phys. A Math. Gen..

[41] Pfeifer P (1993). How fast can a quantum state change with time?. Phys. Rev. Lett..

[42] Margolus N, Levitin LB (1998). The maximum speed of dynamical evolution. Phys. D.

[43] Caneva T (2009). Optimal control at the quantum speed limit. Phys. Rev. Lett..

[44] Bukov M, Sels D, Polkovnikov A (2019). Geometric speed limit of accessible many-body state preparation. Phys. Rev. X.

[45] Jones PJ, Kok P (2010). Geometric derivation of the quantum speed limit. Phys. Rev. A.

[46] Koch J, Le Hur K (2009). Superfluid-Mott-insulator transition of light in the Jaynes-Cummings lattice. Phys. Rev. A.

[47] Houck AA, Türeci HE, Koch J (2012). On-chip quantum simulation with superconducting circuits. Nat. Phys..

[48] Xue J, Seo K, Tian L, Xiang T (2017). Quantum phase transition in a multiconnected Jaynes-Cummings lattice. Phys. Rev. B.

[49] Hoffman AJ (2011). Dispersive photon blockade in a superconducting circuit. Phys. Rev. Lett..

[50] Nissen F (2012). Nonequilibrium dynamics of coupled qubit-cavity arrays. Phys. Rev. Lett..

[51] Fitzpatrick M, Sundaresan NM, Li ACY, Koch J, Houck AA (2017). Observation of a dissipative phase transition in a one-dimensional circuit QED lattice. Phys. Rev. X.

[52] Sala VG (2015). Spin-orbit coupling for photons and polaritons in microstructures. Phys. Rev. X.

[53] Lepert G, Trupke M, Hartmann MJ, Plenio MB, Hinds EA (2011). Arrays of waveguide-coupled optical cavities that interact strongly with atoms. New J. Phys..

[54] Ivanov PA, Ivanov SS, Vitanov NV, Mering A, Fleischhauer M, Singer K (2009). Simulation of a quantum phase transition of polaritons with trapped ions. Phys. Rev. A.

[55] Toyoda K, Matsuno Y, Noguchi A, Haze S, Urabe S (2013). Experimental realization of a quantum phase transition of polaritonic excitations. Phys. Rev. Lett..

[56] Debnath S, Linke NM, Wang S-T, Figgatt C, Landsman KA, Duan L-M, Monroe C (2018). Observation of hopping and blockade of bosons in a trapped ion spin chain. Phys. Rev. Lett..

[57] Li B-W, Mei Q-X, Wu Y-K, Cai M-L, Wang Y, Yao L, Zhou Z-C, Duan L-M (2022). Observation of non-Markovian spin dynamics in a Jaynes-Cummings-Hubbard model using a trapped-ion quantum simulator. Phys. Rev. Lett..

[58] The qubit-cavity coupling $$g(t)$$ and the cavity hopping rate $$J(t)$$ used in our simulation are in dimensionless units. Because the coupling and hopping strengths in superconducting devices are on the order of a few hundreds of MHz, we assume that $$g=1$$ corresponds to $$100$$ MHz when estimating the total evolution time.

[59] Larson, J. & Mavrogordatos, T. The Jaynes-Cummings model and its descendants. arXiv:2202.00330.

[60] Krantz P (2019). A quantum engineer’s guide to superconducting qubits. Appl. Phys. Rev..

[61] Blais A, Grimsmo AL, Girvin SM, Wallraff A (2021). Circuit quantum electrodynamics. Rev. Mod. Phys..

[62] Mitchell BK (2021). Hardware-efficient microwave-activated tunable coupling between superconducting qubits. Phys. Rev. Lett..

[63] Campbell DL, Kamal A, Ranzani L, Senatore L, LaHaye MD (2023). Modular tunable coupler for superconducting circuits. Phys. Rev. Appl..

[64] Chen Y (2014). Qubit architecture with high coherence and fast tunable coupling. Phys. Rev. Lett..

[65] Yan F (2018). Tunable coupling scheme for implementing high-fidelity two-qubit gates. Phys. Rev. Appl..

[66] Sandberg M, Wilson CM, Persson F, Bauch T, Johansson G, Shumeiko V, Duty T, Delsing P (2008). Tuning the field in a microwave resonator faster than the photon lifetime. Appl. Phys. Lett..

