# Supplementary material for: State preparation in a Jaynes-Cummings lattice with quantum optimal control
Source: Sci Rep. 2023 Nov 14;13:19924. doi: 10.1038/s41598-023-47002-1 (PMC10645998; doi:10.1038/s41598-023-47002-1)
Supplement: Supplementary file 1 — Supplementary Information. [file 41598_2023_47002_MOESM1_ESM.pdf]

Supplementary Information for "State Preparation in a Jaynes-Cummings Lattice with Quantum Optimal Control"

Prabin Parajuli,[1] Anuvetha Govindarajan,[1] and Lin Tian[1]
[1]*School of Natural Sciences, University of California, Merced, California 95343, USA*

SUPPLEMENTARY NOTES

Energy Spectrum of JC Model

We consider a single Jaynes-Cummings (JC) model on site $j \in [1, N]$ of the lattice with the Hamiltonian

$$H_{j0} = \left[\omega_c a_j^\dagger a_j + \omega_z \frac{\sigma_{jz}+1}{2} + g\left(a_j^\dagger \sigma_{j-} + \sigma_{j+} a_j\right)\right] \tag{S1}$$

with ω_c the frequency of the cavity mode, a_j ($a_j^\dagger$) the annihilation (creation) operator of the cavity mode, ω_z the energy splitting of the qubit, and $\sigma_{jz}, \sigma_{j\pm}$ the Pauli operators of the qubit. We describe the eigenstates of the JC model using the basis set $\{|n, s\rangle\}$, which are the product states of the photon number state $|n\rangle$ (n being integer) of the cavity and the spin up or down state ($s =\uparrow$ or $\downarrow$) of the qubit.

The eigenstates of the JC model include the ground state $|g_0\rangle = |0, \downarrow\rangle$ with zero photon and the qubit in the spin down state (i.e., no excitation in the model), and the polariton doublets $|n, \pm\rangle$ with n excitations:

$$|n, +\rangle = \cos(\theta/2)|n, \downarrow\rangle + \sin(\theta/2)|n-1, \uparrow\rangle, \tag{S2a}$$

$$|n, -\rangle = \sin(\theta/2)|n, \downarrow\rangle - \cos(\theta/2)|n-1, \uparrow\rangle, \tag{S2b}$$

where $\theta = 2 \arcsin\sqrt{[1-\Delta/\chi(n)]/2}$, $\chi(n) = \sqrt{\Delta^2 + 4ng^2}$, and $\Delta = \omega_c - \omega_z$ is the detuning between the cavity and the qubit [S1].

The eigenenergies for different eigenstates are $E_{g_0} = 0$, and $E_{n,\pm} = n\omega_c - \frac{1}{2}\Delta \pm \frac{\chi(n)}{2}$. When the coupling strength g is nonzero, the energies of the eigenstates are not equally spaced with, e.g., $(E_{n+1,-} - E_{n,-}) > (E_{n,-} - E_{n-1,-})$. This nonlinearity in the energy spectrum is at the root of many interesting phenomena in the JC model (and hence JC lattices), such as the photon blockade effect and the Mott insulator-superfluid phase transition.

QOC Algorithm and Optimization Parameters

In our numerical simulation, we first diagonalize the Hamiltonian of the JC lattice at the initial parameters $g(0)$, $J(0)$ and $\Delta(0)$ to find its ground state $|\psi_0\rangle$. This state is used as the initial state of the evolution with $|\psi(0)\rangle = |\psi_0\rangle$. Here, the initial parameters are chosen so that $|\psi_0\rangle$ is easy to prepare in realistic systems [S2]. We then solve the ground state of the Hamiltonian at the target parameters $g(T)$, $J(T)$ and $\Delta(T)$, which is the desired many-body state $|\psi_{\rm T}\rangle$ to be prepared at the final time T of the evolution.

In our quantum optimal control (QOC) approach, we adopt the chopped random basis (CRAB) algorithm to parameterize the couplings $g(t)$ and $J(t)$ with truncated Fourier series. The parameters $c_{i,k}$, $d_{i,k}$ and $\delta\omega_{i,k}$ ($i = 1, 2$ and $k \in [1, 8]$ being integers), as defined in Eq. (7a) and (7b) in the Methods section of the main paper, include a total of 48 optimization parameters. In the beginning of the optimization process, a random set of $c_{i,k}$, $d_{i,k}$ and $\delta\omega_{i,k}$ are used to generate the initial trial functions for $g(t)$ and $J(t)$. Note that the choice of the random parameters only affects the values of $g(t)$ and $J(t)$ at $0 < t < T$. At $t = 0$ ($t = T$), the couplings always remain the same as the initial (target) parameters of the JC lattice. During the optimization process, following the protocol in Eq. (9) in the Methods section of the main paper, the values of $g(t)$ and $J(t)$ are bounded by the constraints $g_{\max}$ and $J_{\max}$, respectively. Given a set of time-dependent couplings $g(t)$ and $J(t)$, the system evolves from the initial state $|\psi_0\rangle$ under the Hamiltonian $H_t[g(t), J(t)]$ to reach the final state $|\psi(T)\rangle$ at time T. The cost function for this set of couplings can then be calculated. In this work, we use the Nelder-Mead method to minimize the cost function and find the optimal values for $c_{i,k}$, $d_{i,k}$ and $\delta\omega_{i,k}$.

In our simulation, we choose the maximal number of iterations to be 150000. For a given total time T, if the optimization process converges to a fidelity above the threshold value $\mathbb{F}_{\rm th} = 0.99$ within the maximal number of iterations, we consider the QOC process successful. The convergence of the QOC process is determined by the difference between the optimization parameters in adjacent iterations. If the difference is smaller than the pre-defined error tolerance, then we consider that convergence has been achieved. In Fig. S1(a-f), we plot the Fourier coefficients $c_{i,k}$, $d_{i,k}$ and the frequency offsets $\delta\omega_{i,k}$ vs the iteration number n under the constraints $J_{\max} = 1$, $g_{\max} = 2$ and for the total evolution time $T = T_{\rm th} = 3.30\pi$. From the numerical result, we find that the number of iterations for this simulation to converge is $n = 43194$.

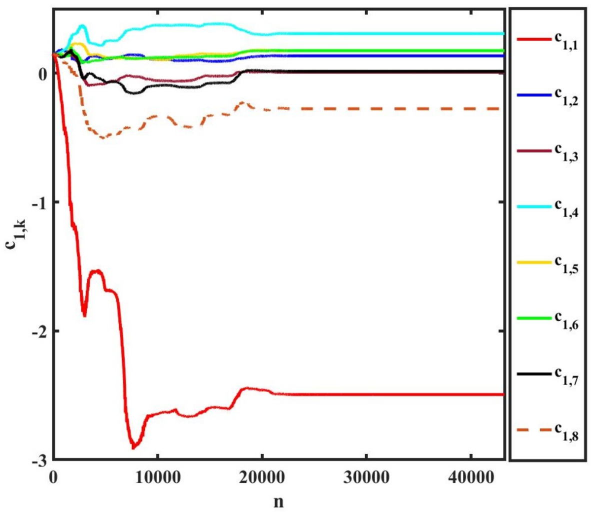

(a) The coefficient $c_{1,k}$ vs the iteration number n.

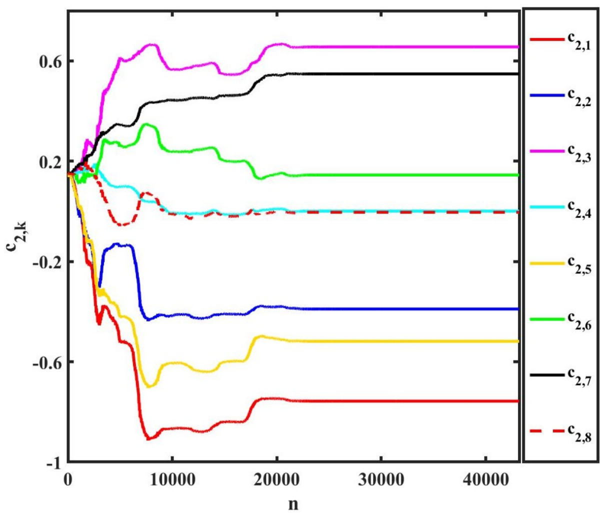

(b) The coefficient $c_{2,k}$ vs the iteration number n.

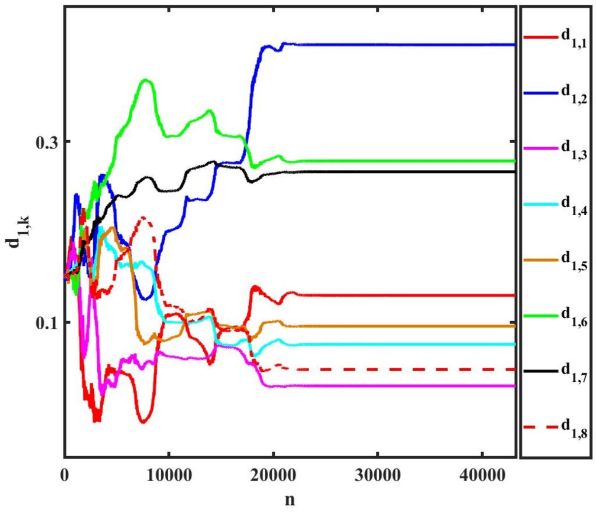

(c) The coefficient $d_{1,k}$ vs the iteration number n.

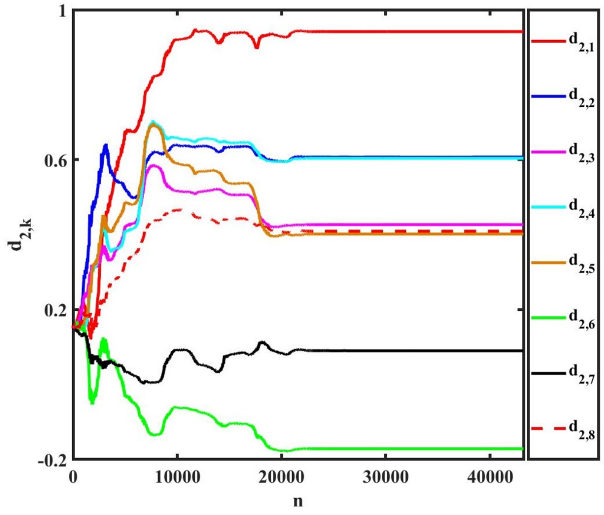

(d) The coefficient $d_{2,k}$ vs the iteration number n.

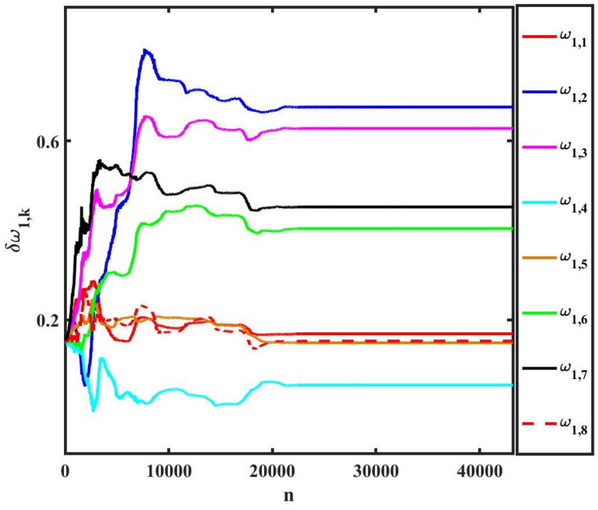

(e) The offset $\delta\omega_{1,k}$ vs the iteration number n.

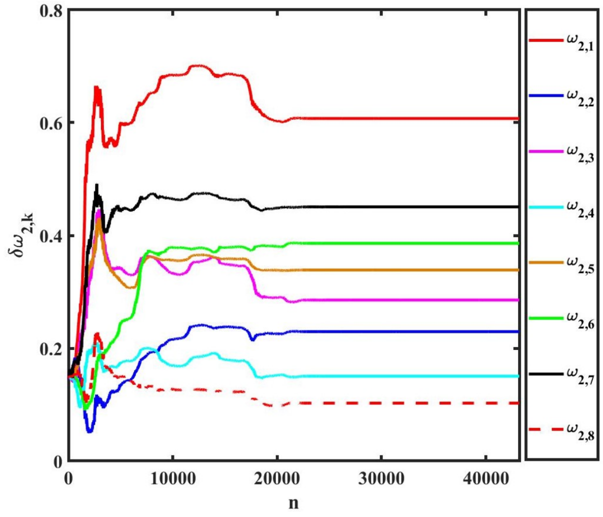

(f) The offset $\delta\omega_{2,k}$ vs the iteration number n.

FIG. S1. Optimization parameters $c_{i,k}$, $d_{i,k}$, and $\delta\omega_{i,k}$ vs the iteration number n. The constraints for the couplings are $J_{\max} = 1$ and $g_{\max} = 2$. The evolution time is $T = T_{\rm th} = 3.30\pi$.

TABLE S1. The fidelity of the prepared state without and with the decoherence terms in Eq. (S3) at $T = 3.3\pi$.

Constraints	$\mathbb{F}$ (w/o decoherence)	$\mathbb{F}$ (with decoherence)
$J_{\max} = 2,\ g_{\max} = 1$	0.8952	0.8937
$J_{\max} = 2,\ g_{\max} = 2$	0.9956	0.9939
$J_{\max} = 2,\ g_{\max} = 4$	0.9994	0.9977

Master Equation Approach for Decoherence

To quantitatively characterize the effect of decoherence, we adopt the following master equation [S3]:

$$\frac{d\rho}{dt} = -i\left[H_t, \rho\right] + \frac{\kappa}{2}\sum_{j=1}^{N}\left(2a_j\rho a_j^\dagger - a_j^\dagger a_j\rho - \rho a_j^\dagger a_j\right) + \frac{\gamma}{2}\sum_{j=1}^{N}\left(2\sigma_{j-}\rho\sigma_{j+} - \sigma_{j+}\sigma_{j-}\rho - \rho\sigma_{j+}\sigma_{j-}\right), \tag{S3}$$

where ρ is the density matrix of the JC lattice, H_t is the total Hamiltonian with the time-dependent, optimized couplings $g(t)$ and $J(t)$, κ is the cavity decay rate, and γ is the qubit decoherence rate. For simplicity of discussion, we assume that all cavity modes have the same decay rate and all qubits have the same decoherence rate. For qubit decoherence, we omit the pure dephasing term that can have the form $\frac{\gamma_d}{2}\sum_{j=1}^{N}(\sigma_{jz}\rho\sigma_{jz} - \rho)$ with dephasing rate γ_d. It can be shown that the dephasing term will have comparable effect as the qubit and cavity decay terms in Eq. (S3).

In the numerical simulation, we use the same parameters as discussed in the main paper. We take the energy unit to be $g = 2\pi \times 100$ MHz, and give all parameters in dimensionless unit according to this energy unit. With a qubit decoherence time of 100μs, the qubit decoherence rate can be derived as $\gamma = 2\pi \times \frac{5}{\pi}$ kHz. In dimensionless unit, this corresponds to a decoherence rate $\gamma = \frac{5}{\pi} \times 10^{-5}$. With a cavity frequency of $\omega_c = 2\pi \times 5$ GHz and a quality factor of $Q = 10^6$, the cavity decay rate is $\kappa = \omega_c/Q = 2\pi \times 5$ kHz. In dimensionless unit, this means $\kappa = 5 \times 10^{-5}$. We run the master equation to test the effect of the decoherence terms with the three sets of parameters used in Fig. 4 of the main paper, i.e., $J_{\max} = 2$, $g_{\max} = 1, 2, 4$, respectively, and $T = 3.3\pi$. The fidelities of the final state without and with the decoherence terms are given in Table. S1. It can be seen that the fidelity is reduced only by ~ 0.0017 in all three cases. This result confirms our analysis in the Discussion section of the main paper that the decoherence effects can be neglected in our QOC approach.

SUPPLEMENTARY REFERENCES

[S1] Blais, A., Huang, R.-S., Wallraff, A., Girvin, S. M. & Schoelkopf, R. J. Cavity quantum electrodynamics for superconducting electrical circuits: An architecture for quantum computation. *Phys. Rev. A* **69**, 062320 (2004).

[S2] Cai, K., Parajuli, P., Long, G.-L., Wong, C.-W. & Tian, L. Robust Preparation of Many-body Ground States in Jaynes-Cummings Lattices. *Npj Quantum Inf.* **7**, 96 (2021).

[S3] D. F. Walls and G. J. Milburn, *Quantum Optics* (Springer, Berlin, Heidelberg, 2008).
